# Engineering the Fab fragment of the anti-IgE omalizumab to prevent Fab crystallization and permit IgE-Fc complex crystallization

**DOI:** 10.1107/S2053230X20001466

**Published:** 2020-03-02

**Authors:** Alkistis N. Mitropoulou, Tom Ceska, James T. Heads, Andrew J. Beavil, Alistair J. Henry, James M. McDonnell, Brian J. Sutton, Anna M. Davies

**Affiliations:** a Randall Centre for Cell and Molecular Biophysics, King’s College London, New Hunt’s House, London SE1 1UL, UK; b Medical Research Council and Asthma UK Centre in Allergic Mechanisms of Asthma, London, UK; c UCB Celltech, 208 Bath Road, Slough SL1 3WE, UK

**Keywords:** omalizumab, allergy, Fab, immunoglobulin E, antibody, protein engineering, X-ray crystallography

## Abstract

Immunoglobulin E (IgE) plays a central role in the allergic response, in which cross-linking of allergen by Fc∊RI-bound IgE triggers mast cell and basophil degranulation and the release of inflammatory mediators. The high-affinity interaction between IgE and Fc∊RI is a long-standing target for therapeutic intervention in allergic disease. Omalizumab is a clinically approved anti-IgE monoclonal antibody that binds to free IgE, also with high affinity, preventing its interaction with Fc∊RI. All attempts to crystallize the pre-formed complex between the omalizumab Fab and the Fc region of IgE (IgE-Fc), to understand the structural basis for its mechanism of action, surprisingly failed. Instead, the Fab alone selectively crystallized in different crystal forms, but their structures revealed intermolecular Fab/Fab interactions that were clearly strong enough to disrupt the Fab/IgE-Fc complexes. Some of these interactions were common to other Fab crystal structures. Mutations were therefore designed to disrupt two recurring packing interactions observed in the omalizumab Fab crystal structures without interfering with the ability of the omalizumab Fab to recognize IgE-Fc; this led to the successful crystallization and subsequent structure determination of the Fab/IgE-Fc complex. The mutagenesis strategy adopted to achieve this result is applicable to other intractable Fab/antigen complexes or systems in which Fabs are used as crystallization chaperones.

## Abstract 2

The omalizumab Fab was engineered to disrupt recurring crystal packing interactions in Fab crystal structures; this led to the eventual structure determination of an omalizumab-derived Fab in complex with its target, IgE-Fc.

## 1. Introduction   

Immunoglobulin E (IgE) plays a central role in allergic disease through the interaction between its Fc region (IgE-Fc) and the Fc∊RI receptor, in which cross-linking of Fc∊RI-bound IgE by allergen triggers mast cell and basophil degranulation, with the release of inflammatory mediators (Gould & Sutton, 2008 ▸).

IgE-Fc, comprising two identical disulfide-linked chains of C∊2, C∊3 and C∊4 domains, adopts a bent conformation in solution (Beavil *et al.*, 1995 ▸; Davis *et al.*, 1990 ▸; Holowka & Baird, 1983 ▸; Holowka *et al.*, 1985 ▸; Hunt *et al.*, 2012 ▸; Zheng *et al.*, 1991 ▸, 1992 ▸). In the crystal structure of unbound IgE-Fc, the Fc region is acutely bent: the (C∊2)_2_ domain pair folds back against the C∊3 and C∊4 domains, with an angle of 62° between the local twofold axes of the C∊2 and C∊4 domain pairs (Doré *et al.*, 2017 ▸; Holdom *et al.*, 2011 ▸; Wan *et al.*, 2002 ▸). The Fc∊3-4 region, comprising only the C∊3 and C∊4 domains, is conformationally flexible, and the C∊3 domains can adopt a variety of positions relative to one another, from ‘closed’ to ‘open’ (Chen *et al.*, 2018 ▸; Cohen *et al.*, 2014 ▸; Davies *et al.*, 2017 ▸; Dhaliwal *et al.*, 2012 ▸, 2014 ▸, 2017 ▸; Doré *et al.*, 2017 ▸; Drinkwater *et al.*, 2014 ▸; Garman *et al.*, 2000 ▸; Holdom *et al.*, 2011 ▸; Jabs *et al.*, 2018 ▸; Wan *et al.*, 2002 ▸; Wurzburg & Jardetzky, 2009 ▸; Wurzburg *et al.*, 2000 ▸; Yuan *et al.*, 2013 ▸), a property associated with the mutually exclusive, allosteric regulation of binding to Fc∊RI and the second principal receptor for IgE, CD23 (Borthakur *et al.*, 2012 ▸; Dhaliwal *et al.*, 2012 ▸). The C∊3 domains adopt an open conformation, and IgE-Fc becomes more acutely bent, when in complex with Fc∊RI (Garman *et al.*, 2000 ▸; Holdom *et al.*, 2011 ▸; Hunt *et al.*, 2012 ▸), while CD23 binds when the C∊3 domains adopt a closed conformation (Dhaliwal *et al.*, 2012 ▸, 2014 ▸, 2017 ▸; Yuan *et al.*, 2013 ▸). The potential for more extreme flexibility in IgE-Fc was first revealed when a fully extended, linear structure, involving a ∼120° unbending of the (C∊2)_2_ domain pair relative to the Fc∊3-4 region, was captured by an anti-IgE Fab (Drinkwater *et al.*, 2014 ▸). Molecular dynamics simulations have also revealed that IgE-Fc can adopt relatively stable, more extended conformations, between the two extremes of acutely bent and fully extended (Drinkwater *et al.*, 2014 ▸).

The high-affinity interaction between IgE and Fc∊RI is a long-standing target in the development of treatments for allergic disease (Holgate, 2014 ▸). Omalizumab is an anti-IgE therapeutic monoclonal IgG1 antibody that inhibits the interaction of IgE with Fc∊RI and is approved for the treatment of moderate-to-severe persistent allergic asthma and chronic idiopathic urticaria (Holgate *et al.*, 2005 ▸; Sussman *et al.*, 2014 ▸). Although the binding site for omalizumab had previously been mapped to the C∊3 domain (Zheng *et al.*, 2008 ▸), and omalizumab was known to bind to a partially bent IgE-Fc conformation (Hunt *et al.*, 2012 ▸), the structural basis for its mechanism of action was poorly understood until only recently.

We, and others (Jensen *et al.*, 2015 ▸), had attempted to crystallize the complex between the omalizumab Fab and IgE-Fc. However, despite extensive efforts, our crystallization trials of pre-formed omalizumab Fab/IgE-Fc and Fc∊3-4 complexes only resulted in selective crystallization of the Fab. The structure of the omalizumab Fab in complex with the Fc∊3-4 region of IgE-Fc has been reported, which revealed details of the omalizumab epitope on the C∊3 domain (Pennington *et al.*, 2016 ▸). However, this Fc∊3-4 molecule lacked the (C∊2)_2_ domain pair and was conformationally constrained by an engineered disulfide bond that locked the C∊3 domains into a closed conformation (Pennington *et al.*, 2016 ▸). Given the flexible nature of the Fc∊3-4 region, and the potential for extreme flexibility in IgE-Fc, which additionally contains the (C∊2)_2_ domain pair, this structure could thus provide only limited mechanistic insights.

We designed a mutagenesis strategy to disrupt the packing interactions observed in omalizumab Fab crystal structures, without affecting the antigen-binding CDRs, with the aim of crystallizing the complex between the omalizumab Fab and IgE-Fc. The strategy first involved creating a point mutation in a short segment of β-strand structure found in the Cκ domain CD loop, followed by two point mutations in the V_L_ domain EF loop.

One omalizumab-derived Fab, termed FabXol3, which contains three point mutations in the light chain, subsequently enabled us to solve the 3.7 Å resolution crystal structure of the complex with IgE-Fc, revealing that omalizumab inhibits binding to Fc∊RI allosterically (Davies *et al.*, 2017 ▸). In this complex, IgE-Fc adopts a partially bent conformation, and the C∊3 domains adopt a markedly open conformation, more open than that seen in any other crystal structure thus far.

Here, we report the structural basis and rationale for this mutagenesis strategy. Such an approach could inform the design and structure determination of other Fabs in complex with their target proteins in cases where the pre-formed complex is disrupted by the selective crystallization of one partner, in particular the Fab.

## 2. Materials and methods   

### 2.1. Macromolecule production   

IgE-Fc, Fc∊3-4, FabXol, FabXol2, FabXol3 and scFvXol proteins were produced using previously described methods (Davies *et al.*, 2017 ▸; Dhaliwal *et al.*, 2012 ▸; Drinkwater *et al.*, 2014 ▸; Weatherill *et al.*, 2012 ▸; Young *et al.*, 1995 ▸). Omalizumab was purchased from Novartis Europharm Ltd.

### 2.2. Crystallization   

All crystals were grown at 18°C using the sitting-drop vapor-diffusion method in MRC 96-well plates. FabXol^1^ and FabXol^2^ (omalizumab Fab) crystals were grown from un­successful crystallization trials of the FabXol/IgE-Fc and FabXol/Fc∊3-4 complexes. For the FabXol^1^ and FabXol^2^ structures reported here, the 2:1 complex between FabXol and Fc∊3-4 was purified by size-exclusion chromatography, buffer-exchanged into 25 m*M* Tris–HCl pH 7.5, 20 m*M* NaCl and concentrated to 18.8 mg ml^−1^. FabXol^1^ crystals were grown in 0.085 *M* Tris pH 8.5, 42.5%(*v*/*v*) MPD, 15%(*v*/*v*) glycerol, 0.17 *M* ammonium phosphate and were cryoprotected with the mother liquor. FabXol^2^ crystals were grown in 0.1 *M* phosphate–citrate pH 4.2, 20%(*w*/*v*) PEG 1000, 0.2 *M* lithium sulfate and were cryoprotected with 0.1 *M* sodium acetate pH 4.6, 25%(*w*/*v*) PEG 4000, 18%(*v*/*v*) ethylene glycol. For both crystals, a reservoir volume of 50 µl was used and the drops consisted of 100 nl protein solution and 200 nl reservoir solution.

FabXol1^1^ and FabXol1^2^ (omalizumab-derived Leu158Pro light-chain mutant Fab) crystals were grown from unsuccessful crystallization trials of the FabXol1/IgE-Fc complex. The 2:1 complex between FabXol1 and IgE-Fc was purified by size-exclusion chromatography, buffer-exchanged into 0.25 *M* Tris–HCl pH 7.5, 0.2 *M* NaCl and concentrated to 18.8 mg ml^−1^. FabXol1^1^ crystals were grown in 20%(*w*/*v*) PEG 3350, 0.2 *M* sodium sulfate and were cryoprotected with 20%(*w*/*v*) PEG 3350, 0.2 *M* magnesium sulfate, 18%(*v*/*v*) ethylene glycol. FabXol1^2^ crystals were grown in 20%(*w*/*v*) PEG 4000, 0.2 *M* magnesium sulfate, 10%(*v*/*v*) glycerol and were cryoprotected in 20%(*w*/*v*) PEG 4000, 0.2 *M* magnesium sulfate, 18%(*v*/*v*) glycerol. For both crystals, a reservoir volume of 50 µl was used and the drops consisted of 100 nl protein solution and 200 nl reservoir solution.

FabXol2 (omalizumab-derived Ser81Arg, Gln83Arg light-chain mutant Fab) was buffer-exchanged into 0.1 *M* Tris–HCl pH 8.5, 0.05 *M* NaCl and concentrated to 3 mg ml^−1^. FabXol2 crystals were grown in 0.1 *M* HEPES pH 7, 20%(*w*/*v*) PEG 4000 and were cryoprotected with 12%(*v*/*v*) PEG 400, 17%(*v*/*v*) glycerol; a reservoir volume of 100 µl was used and the drops consisted of 200 nl protein solution and 100 nl reservoir solution. FabXol3 (omalizumab-derived Ser81Arg, Gln83Arg, Leu158Pro light-chain mutant Fab) was purified in phosphate-buffered saline (PBS), concentrated to 15 mg ml^−1^ and then diluted to 5 mg ml^−1^ with 0.1 *M* Tris pH 8.5. FabXol3 crystals were grown in 0.1 *M* HEPES pH 7, 20%(*w*/*v*) PEG 4000, 0.15 *M* ammonium sulfate and were cryoprotected with 0.1 *M* HEPES pH 7.5, 20%(*w*/*v*) PEG 4000, 0.1 *M* ammonium sulfate, 15%(*v*/*v*) ethylene glycol; a reservoir volume of 50 µl was used and the drops consisted of 100 nl protein solution and 200 nl reservoir solution. scFvXol was buffer-exchanged into 0.25 *M* Tris–HCl pH 8.5, 0.2 *M* NaCl and concentrated to 3.9 mg ml^−1^. scFvXol crystals were grown in 0.1 *M* trisodium citrate pH 5.6, 15%(*w*/*v*) PEG 4000, 0.2 *M* ammonium sulfate and were cryoprotected with 0.1 *M* trisodium citrate pH 5.6, 30%(*w*/*v*) PEG 4000, 0.2 *M* ammonium sulfate; a reservoir volume of 100 µl was used and the drops consisted of 100 nl protein solution and 80 nl reservoir solution.

### 2.3. X-ray data collection, processing, structure determination and refinement   

Data were collected on beamlines I02, I03, I04, I04-1 and I24 at the Diamond Light Source, Harwell, UK. Data were integrated with *XDS* (Kabsch, 2010 ▸) using the *xia*2 package (Winter, 2010 ▸) or with *MOSFLM* (Leslie & Powell, 2007 ▸), and were scaled with *AIMLESS* (Evans & Murshudov, 2013 ▸) or *SCALA* (Evans, 2006 ▸) from the *CCP*4 suite (Winn *et al.*, 2011 ▸). Structures were solved by molecular replacement using *MOLREP* (Vagin & Teplyakov, 2010 ▸) or *Phaser* (McCoy *et al.*, 2007 ▸). Protein atoms from PDB entry 2fjf (Fuh *et al.*, 2006 ▸) were used as a search model for the FabXol^1^ structure. Subsequent structures were solved using protein atoms (V_H_, V_L_, Cκ and Cγ1 domains) from the FabXol^1^ structure as a search model, although the CDR residues were removed. The structures were initially refined with *REFMAC* (Murshudov *et al.*, 2011 ▸) and subsequently with *Phenix* (Liebschner *et al.*, 2019 ▸), and refinement was alternated with rounds of manual model building with *Coot* (Emsley *et al.*, 2010 ▸). Model quality was assessed with *MolProbity* (Chen *et al.*, 2010 ▸). Data-processing and refinement statistics are summarized in Tables 1 ▸ and 2 ▸. Interfaces were analyzed with *PISA* (Krissinel & Henrick, 2007 ▸). Figures were produced with *PyMOL*.

### 2.4. PDB references   

Coordinates and structure factors have been deposited in the Protein Data Bank with the following accession codes: FabXol^1^, 6tcm; FabXol^2^, 6tcn; FabXol1^1^, 6tco; FabXol1^2^, 6tcp; FabXol2, 6tcq; FabXol3, 6tcr; scFvXol, 6tcs.

### 2.5. Fluorescence-based thermal stability (*T*_m_) measurement   

A thermal stability assay was performed using a QuantStudio 7 Real-Time PCR System (Thermo Fisher). 5 µl of 30× SYPRO Orange Protein Gel Stain (Thermo Fisher), diluted from 5000× concentrate with PBS pH 7.4, was added to 45 µl protein sample (0.2 mg ml^−1^ in PBS pH 7.4) and mixed. 10 µl of this solution was dispensed into an optical 384-well PCR plate. The PCR heating device was set at 20°C and increased to 99°C at a rate of 1.1°C min^−1^. A charge-coupled device was used to monitor fluorescence changes in the wells. Fluorescence intensity increases were plotted and the inflection point of the slope was used to generate apparent midpoint temperatures (*T*_m_).

### 2.6. Surface plasmon resonance   

Surface plasmon resonance binding experiments were performed using a Biacore T200 instrument (GE Healthcare). Intact omalizumab, the Fabs and scFv were immobilized at similar densities on CM5 sensor chips using an amine-coupling protocol according to the manufacturer’s instructions (GE Healthcare). The following immobilization densities were used for these studies: omalizumab, 970 resonance units; FabXol, 200 resonance units; FabXol2, 270 resonance units; FabXol3, 210 resonance units; scFvXol, 250 resonance units. For binding studies, IgE-Fc, in a twofold dilution series (100–0.4 n*M*), was injected at a flow rate of 20 µl min^−1^ for 240 s, followed by a dissociation time of 900 s. All binding experiments were performed at 25°C in 20 m*M* HEPES pH 7.4, 150 m*M* NaCl, 0.005%(*v*/*v*) surfactant P20. *BIAevaluation* (GE Healthcare) and *Origin* 8 (OriginLab) were used to analyze and present the data. For a visual comparison of IgE-Fc binding curves to the different omalizumab constructs, the 100 n*M* concentration for each was adjusted to give a maximal binding of 100 resonance units and these curves were overlaid.

## 3. Results   

The nomenclature used for the omalizumab-derived Fabs and scFv reported here, and their crystal structures, is presented in Table 3 ▸. Heavy- and light-chain CDRs are defined as follows: CDRH1, Ser25–Asn36; CDRH2, Ser51–Asn59; CDRH3, Ala97–Val110; CDRL1, Arg24–Asn38; CDRL2, Tyr53–Ser60; CDRL3, Gln93–Thr101 (North *et al.*, 2011 ▸).

### 3.1. Crystal structures of FabXol (wild-type omalizumab Fab): FabXol^1^ and FabXol^2^   

The structure of FabXol (wild-type omalizumab Fab) was solved in two different crystal forms, which have also been reported by others (Jensen *et al.*, 2015 ▸; Wright *et al.*, 2015 ▸), and the space groups and unit-cell parameters of these structures, FabXol^1^ and FabXol^2^, the latter now reported at a substantially higher resolution, are provided in Table 1 ▸. The structures reported here were the result of unsuccessful crystallization trials of the complex between FabXol and an unconstrained Fc∊3-4 molecule, but similar crystals were also grown from crystallization trials of FabXol in complex with IgE-Fc.

**Figure 1:**
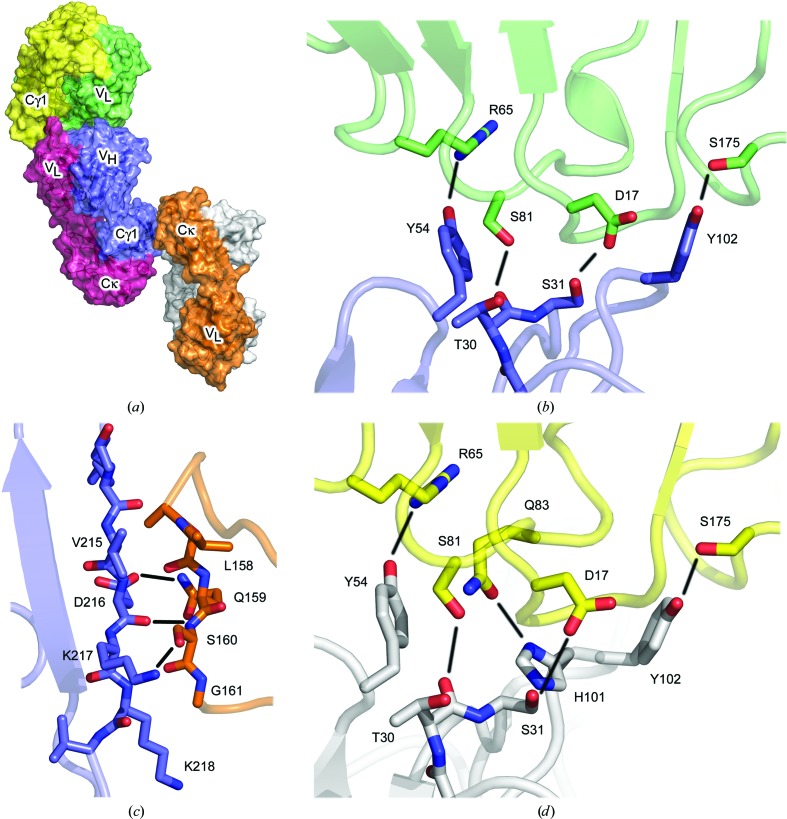
Structure of the omalizumab Fab (FabXol). (*a*) The FabXol^1^ structure contains one Fab molecule (pink and blue) in the asymmetric unit. The heavy-chain CDRs of this Fab contact the V_L_ and Cκ domains (the latter hidden in this view) of one symmetry-related molecule (green and yellow) and the Cκ domain of another (orange and gray). (*b*) Interface between heavy-chain CDR residues (blue) and V_L_ and Cκ domain framework residues from a symmetry-related molecule (green) in the FabXol^1^ structure. Hydrogen bonds are depicted by black lines. (*c*) Interface between an edge β-strand from the Cγ1 domain (blue) and the Cκ domain from a symmetry-related molecule (orange) in the FabXol^1^ structure. Hydrogen bonds are depicted by black lines. (*d*) Interface between heavy-chain CDR residues (gray) and V_L_ and Cκ domain framework residues from a symmetry-related molecule (yellow) for FabXol^2*B*^, which includes a hydrogen bond between His101 (CDRH3) and Gln81 (V_L_ domain). Hydrogen bonds are depicted by black lines.

The FabXol^1^ structure (1.85 Å resolution) contains one Fab in the asymmetric unit, which forms two distinct interfaces with symmetry-related molecules (Fig. 1 ▸ *a*). In the first interface, with an area of ∼395 Å^2^, residues from all three heavy-chain CDRs contact V_L_ and Cκ domain framework residues from a symmetry-related molecule; namely, the V_L_ domain AB, C′′D and EF loops, and the Cκ domain DE loop. In addition to van der Waals interactions, this interface comprises four hydrogen bonds, namely Thr30 (CDRH1)–Ser81 (V_L_), Ser31 (CDRH1)–Asp17 (V_L_), Tyr54 (CDRH2)–Arg65 (V_L_) and Tyr102 (CDRH3)–Ser175 (Cκ) (Fig. 1 ▸ *b*).

The second interface, with an area of ∼324 Å^2^, includes an extensive network of hydrogen bonds between an edge β-strand from the Cγ1 domain (β-strand G) and a short segment of β-strand structure in the Cκ domain CD loop from a symmetry-related molecule. Here, the β-strands are arranged in a parallel manner, with hydrogen bonds between the main-chain atoms of Lys214–Lys218 (Cγ1) and Leu158–Ser160 (Cκ), and between the side chains of Lys217 (Cγ1) and Ser160 (Cκ) (Fig. 1 ▸ *c*). This interface is repeated throughout the crystal lattice, as an identical interface forms between Leu158–Ser160 (Cκ) and Lys214–Lys218 (Cγ1) from a symmetry-related molecule.

The FabXol^2^ structure (2.3 Å resolution) contains two Fab molecules in the asymmetric unit, which are referred to here as FabXol^2*A*^ and FabXol^2*B*^. The CDRs of both molecules adopt similar conformations to those observed in the FabXol^1^ structure. CDRH1–3 residues also interact with the V_L_ and Cκ domain framework residues, akin to the first interface observed in the FabXol^1^ structure, which for FabXol^2*B*^ also includes a hydrogen bond between His101 (CDRH3) and Gln83 (V_L_) (Fig. 1 ▸ *d*). The arrangement of Fabs in the FabXol^2^ asymmetric unit precludes the propagation of the second, β-strand-mediated interface throughout the crystal lattice by a single Fab molecule, as in the FabXol^1^ structure. However, interactions between FabXol^2*A*^ and FabXol^2*B*^, and different symmetry-related molecules, each display this same β-strand interaction, in which Lys214–Lys218 (Cγ1) from FabXol^2A^ interact with Leu158–Ser160 (Cκ) from one symmetry-related molecule, while Leu158–Ser160 (Cκ) from FabXol^2*B*^ interact with Lys214–Lys218 (Cγ1) from a different symmetry-related molecule.

### 3.2. Crystal structure of scFvXol (omalizumab-derived scFv)   

We also attempted to crystallize the complex between a single-chain form of omalizumab (scFvXol) and IgE-Fc, but were unsuccessful. However, we solved the crystal structure of scFvXol alone, in which the light- and heavy-chain variable domains are connected by a (Gly_4_Ser)_4_ linker, to 2.3 Å resolution (Table 1 ▸). The scFvXol structure contains one molecule in the asymmetric unit.

In this structure, the β-strand-mediated crystal packing interaction observed in the FabXol^1^ and FabXol^2^ structures is absent, as the construct lacks the Cγ1 and Cκ domains. However, CDRH1–3 residues from a symmetry-related molecule contact the V_L_ domain of scFvXol in a similar manner to the first interface described for the FabXol^1^ and FabXol^2^ structures, although the interface area is reduced from ∼395 to ∼290 Å^2^ due to the absence of the Cκ domain in scFvXol.

### 3.3. Mutagenesis strategy I: disrupting the interaction between the Cγ1 and Cκ domains   

Crystallization trials of the complexes between FabXol (omalizumab Fab) and IgE-Fc, between scFvXol (omalizumab-derived scFv) and IgE-Fc, and between FabXol and an unconstrained Fc∊3-4 molecule all led to selective crystallization of the Fab or were unsuccessful. Two recurring interfaces in the Fab and scFvXol structures, described in Section 3.1, suggested a route to disrupt crystal packing interactions without mutating the CDR residues responsible for IgE-Fc binding.

We first attempted to disrupt the interface between the edge β-strand (β-strand G) from the Cγ1 domain (Lys214–Lys218) and the short β-strand segment in the Cκ domain CD loop (Leu158–Ser160), observed in the FabXol^1^ and FabXol^2^ structures. Leu158 from the Cκ domain CD loop was mutated to proline, with the aim of altering its secondary structure, to disrupt the extensive, hydrogen-bond-mediated interactions. This omalizumab-derived Leu158Pro mutant Fab was termed FabXol1.

### 3.4. Crystal structures of FabXol1 (omalizumab-derived Leu158Pro mutant Fab): FabXol1^1^ and FabXol1^2^   

The Leu158Pro mutation alone was not sufficient to prevent selective crystallization of the Fab, and the structures reported here were the result of unsuccessful crystallization trials of the complex between FabXol1 and IgE-Fc. Two structures were solved for FabXol1, in new crystal forms, and the space groups and unit-cell parameters of these structures, FabXol1^1^ and FabXol1^2^, are provided in Table 2 ▸.

**Figure 2:**
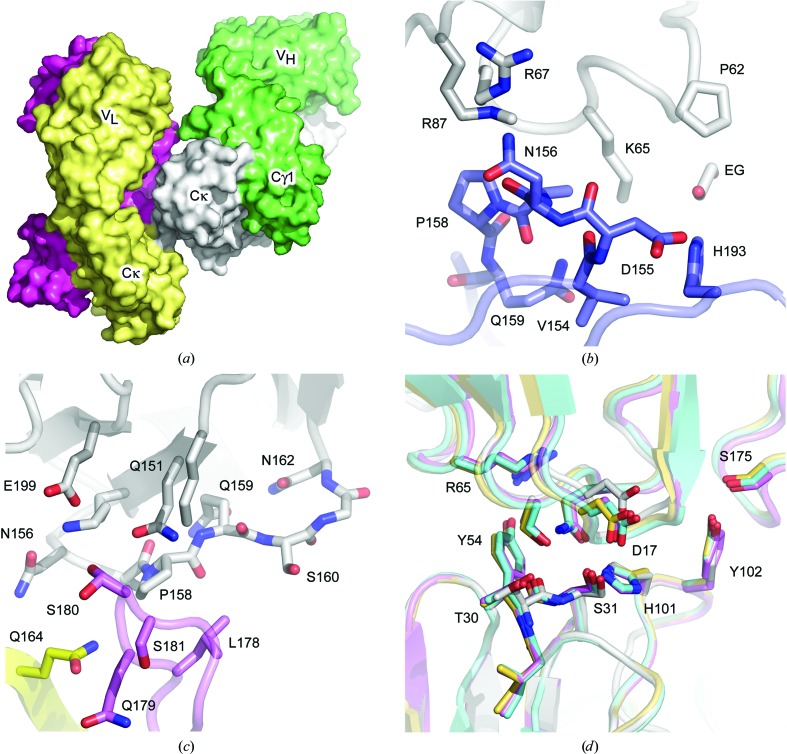
Structure FabXol1^1^ of the omalizumab-derived Leu158Pro mutant (FabXol1). (*a*) The FabXol1^1^ structure contains two molecules (pink and yellow/green and gray) in the asymmetric unit. (*b*) Interface between residues 155–159 of the Cκ domain (blue) of molecule FabXol1^1*A*^ and the V_H_ domain of a symmetry-related molecule (gray). An ethylene glycol molecule (EG) is also bound at this interface. (*c*) Interface between the Cκ domain (gray) of molecule FabXol1^1*B*^ and the Cκ domain (yellow) and Cγ1 domain (pink) of the noncrystallographic symmetry-related molecule, FabXol1^1*A*^. (*d*) Conformations for the CDRH1–3 residues, and their crystal packing interactions with the V_L_ domain (and Cκ domain in the Fabs), are similar for FabXol^1^ (pink), FabXol1^1*A*^ (yellow), FabXol1^1*B*^ (blue) and scFvXol (gray).

The FabXol1^1^ structure (1.8 Å resolution) contains two Fab molecules (FabXol1^1*A*^ and FabXol1^1*B*^) in the asymmetric unit (Fig. 2 ▸ *a*). In this structure, the network of hydrogen bonds observed in the FabXol structures between β-strands of the Cγ1 and Cκ domains is indeed disrupted, but the engineered residue, Pro158, now forms other crystal packing interactions.

In molecule FabXol1^1*A*^, Asp155–Gln159, and His193 (Cκ), including Pro158, form an interface with Pro62, Lys65–Arg67 and Arg87 (V_H_) from a crystallographic symmetry-related molecule, burying a surface area of 187 Å^2^ (Fig. 2 ▸ *b*). In molecule FabXol1^1*B*^, Lys149, Gln151, Lys153, Asn156, Pro158–Gly161, and Glu199 (Cκ), form an interface of 215 Å^2^ with Gly161, Ser163 and Gln164 (Cκ), Ala88 and Glu89 (V_H_), and Leu178–Gly182 (Cγ1) from the noncrystallographic symmetry-related molecule FabXol1^1*A*^ (Fig. 2 ▸ *c*).

CDRH1–3 residues in both molecules of the FabXol1^1^ structure adopt essentially identical conformations to those found in the FabXol^1^ and FabXol^2^ (wild-type omalizumab Fab) and scFvXol (omalizumab-derived scFv) structures. They form similar crystal packing interactions to the first interface described for the FabXol^1^ structure, in which the heavy-chain CDRs contact the V_L_ domain AB, C′′D and EF loops, and the Cκ domain DE loop from a symmetry-related molecule. In both molecules, hydrogen bonds form between Ser31 (CDRH1) and Asp17 (V_L_), between Tyr54 (CDRH2) and Arg65 (V_L_) and between Tyr102 (CDRH3) and Ser175 (Cκ) (Fig. 2 ▸ *d*).

**Figure 3:**
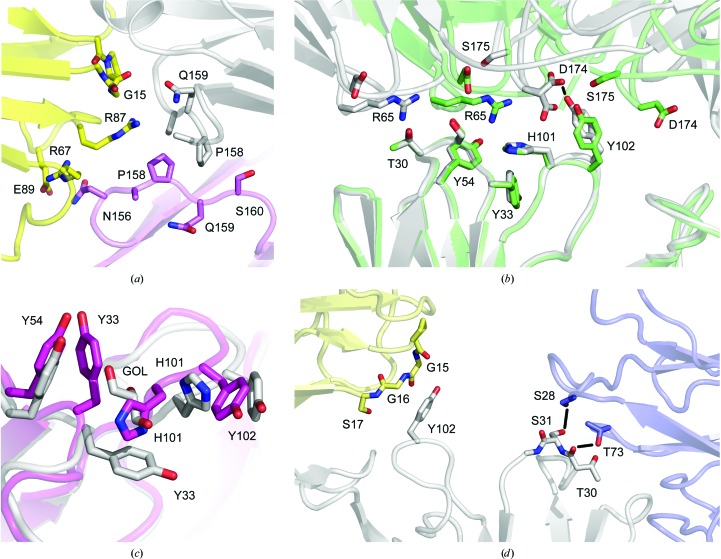
Structure FabXol1^2^ of the omalizumab-derived Leu158Pro mutant (FabXol1). (*a*) In FabXol1^2*C*^ (pink), residues Pro158–Ser160 form an interface with the Cκ domain from FabXol1^2*A*^ (gray) and the V_H_ domain from FabXol1^2*B*^ (yellow). (*b*) The FabXol1^2*A*^ (gray) and FabXol1^2*B*^ (green) CDRs adopt similar conformations, and both contact the V_L_ and Cκ domains from a symmetry-related molecule. A shift in the position of the symmetry-related molecule relative to FabXol1^2*A*^ reduces the interface area, and only a single hydrogen bond is formed between Tyr102 (CDRH3) and Asp174 (Cκ domain). (*c*) Binding of a glycerol molecule (GOL) in FabXol1^2*C*^ (gray) causes the Tyr33 and His101 side chains to adopt substantially different positions compared with those in FabXol1 (pink). (*d*) In FabXol1^2*C*^ (gray), Thr30 and Ser31 form hydrogen bonds with Thr73 and Ser28, respectively, from a symmetry-related molecule (blue). Tyr102 packs against Gly15 and Gly16 from a different symmetry-related molecule (yellow).

The FabXol1^2^ structure (2.5 Å resolution) contains four Fab molecules (FabXol1^2*A*^–FabXol1^2*D*^) in the asymmetric unit. In this structure, the packing environment of Pro158 differs from that in the FabXol1^1^ structure. Again, the β-strand inter­actions between Cγ1 and Cκ domains are disrupted, but new packing interactions involving Pro158 are formed. In all four molecules of the FabXol1^2^ structure, Pro158 forms van der Waals inter­actions with Pro158–Ser160 (Cκ) from a noncrystallographic symmetry-related Fab (Fig. 3 ▸ *a*). In this manner, Pro158 mediates light-chain/light-chain interactions between FabXol1^2*A*^ and FabXol1^2*C*^, and between FabXol1^2*B*^ and FabXol1^2*D*^. Due to the arrangement of the four Fab molecules in the asymmetric unit, Pro158 from FabXol1^2*C*^ is positioned at an interface comprising three Fabs (FabXol1^2*A*^–FabXol1^2*C*^), and in addition to the interface with Pro158–Ser160 from FabXol1^2*A*^, also contacts Arg87 (V_H_) from FabXol1^2*B*^ (Fig. 3 ▸ *a*).

In molecules FabXol1^2*A*^ and FabXol1^2*B*^, the heavy-chain CDRs adopt similar conformations to those in the FabXol, scFvXol and FabXol1^1^ structures. CDR residues from FabXol1^2*B*^ form a similar interface with V_L_ and Cκ domain framework residues from a symmetry-related molecule; hydrogen bonds form between Ser31 (CDRH1) and Asp17 (V_L_), between Tyr54 (CDRH2) and Arg65 (V_L_), between His101 (CDRH3) and Gln83 (V_L_) and between Tyr102 (CDRH3) and Ser175 (Cκ), burying a surface area of 384 Å^2^. Although FabXol1^2*A*^ contacts the V_L_ and Cκ domains of a symmetry-related molecule, the position of this molecule is shifted and the interface area, which is reduced to 274 Å^2^, contains a single hydrogen bond between Tyr102 (CDRH3) and Asp174 (Cκ) (Fig. 3 ▸ *b*).

By contrast, the CDRH1 and CDRH3 conformations differ in molecules FabXol1^2*C*^ and FabXol1^2*D*^ compared with the other structures described thus far. In these molecules, binding of a glycerol molecule causes the Tyr33 (CDRH1) and His101 (CDRH3) side chains to adopt substantially different positions (Fig. 3 ▸ *c*), the implications of which are discussed later. Crystal contacts for FabXol1^2*C*^ and FabXol1^2*D*^ also differ markedly compared with the other Fabs. In FabXol1^2*C*^, Thr30 and Ser31 (CDRH1) form hydrogen bonds with Thr73 and Ser28 (V_L_), respectively, from one symmetry-related molecule, while Tyr102 (CDRH3) packs against Gly15 and Gly16 (V_H_) from another molecule (Fig. 3 ▸ *d*). On the other hand, in FabXol1^2*D*^, only the interaction between Tyr102 and Gly15 and Gly16 from the second symmetry-related molecule is found; the first molecule is positioned further away, precluding hydrogen bonds between Thr30 (CDRH1) and Thr73, and between Ser31 (CDRH1) and Ser28. By contrast, CDRH2 residues do not participate in any crystal contacts, and adopt similar conformations to those in FabXol1^2*A*^ and FabXol1^2*B*^.

Despite the different contacts formed by CDRH1 and CDRH3 in molecules FabXol1^2*C*^ and FabXol1^2*D*^, the packing environment would not preclude the CDR conformations observed in the FabXol, scFvXol and FabXol1^1^ structures, and in molecules FabXol1^2*A*^ and FabXol1^2*B*^.

### 3.5. Mutagenesis strategy II: disrupting packing interactions involving the heavy-chain CDRs   

Although the Leu158Pro mutation in the short β-strand segment of the Cκ domain CD loop disrupted the interaction with the Cγ1 domain edge β-strand (strand G), it did not prevent selective crystallization of the Fab. We next attempted to disrupt the interface between the heavy-chain CDRs and the V_L_ and Cκ domain framework residues. As most of this interface involves interactions between the CDRs and the V_L_ domain, and mutating the CDRs could adversely affect the interaction with IgE-Fc, we mutated Ser81 and Gln83 from the V_L_ domain EF loop, which contribute to this interface, to Arg81 and Arg83, respectively, thus incorporating bulkier, charged side chains. We created two omalizumab-derived Fabs, namely FabXol2, with Ser81Arg and Gln83Arg mutations, and FabXol3, which additionally contains a Leu158Pro mutation. Thermal stability measurements revealed that the incorporation of these three point mutations, either alone or in combination with one another, did not substantially affect the overall stability of the Fabs (Table 4 ▸).

### 3.6. Crystal structures of FabXol2 (omalizumab-derived Ser81Arg, Gln83Arg mutant Fab) and FabXol3 (omalizumab-derived Ser81Arg, Gln83Arg, Leu158Pro mutant Fab)   

Complexes between IgE-Fc and both of the omalizumab-derived Fabs that contained the Ser81Arg and Gln83Arg mutations were eventually crystallized. Crystals with a similar morphology were grown for each complex, although the FabXol3/IgE-Fc complex crystals diffracted to higher resolution, and we recently reported the crystal structure of the complex to 3.7 Å resolution (Davies *et al.*, 2017 ▸).

To understand the effects of the Ser81Arg and Gln83Arg (V_L_) mutations on Fab crystal packing interactions, we solved the structures of FabXol2 and FabXol3 alone. Both FabXol2 and FabXol3 crystallized in the same crystal form (Table 2 ▸), with one Fab molecule in the asymmetric unit. With the exception of the light-chain residue 158, which is leucine in FabXol2 and proline in FabXol3, the structures are otherwise essentially identical.

**Figure 4:**
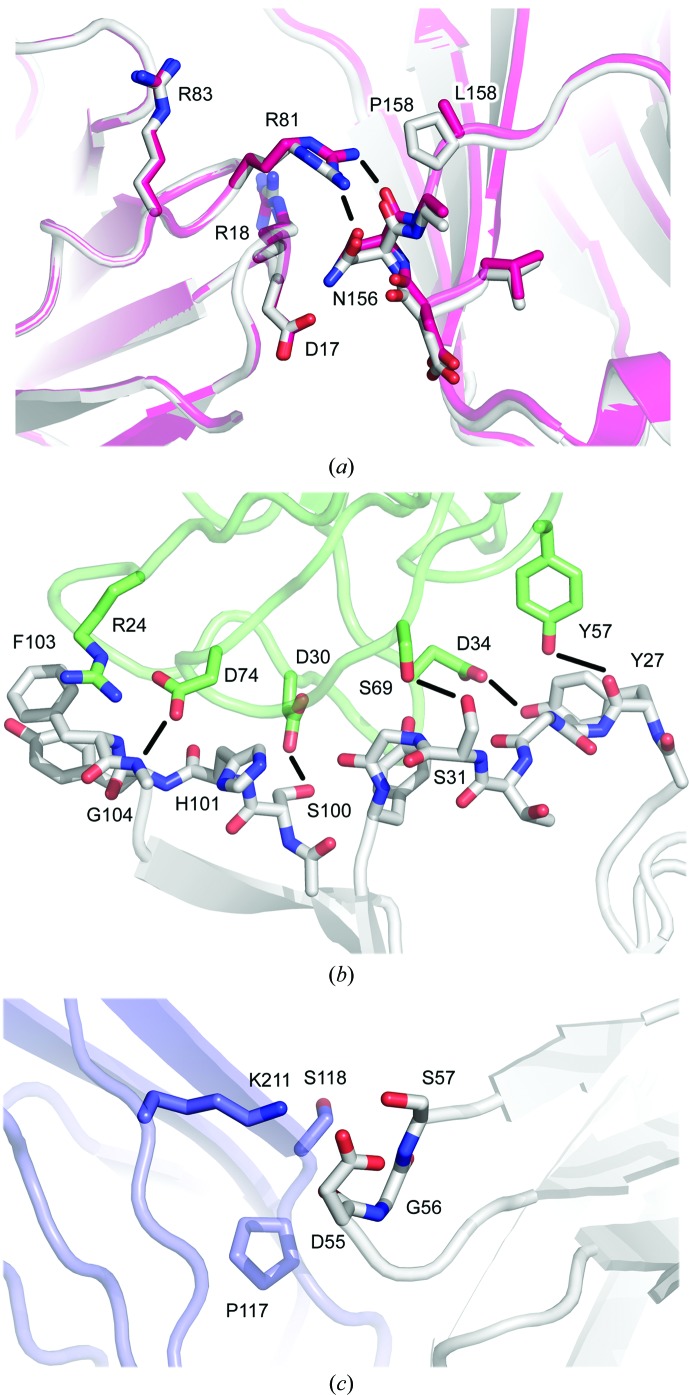
Structure of the omalizumab-derived Ser81Arg, Gln83Arg, Leu158Pro mutant (FabXol3). (*a*) In the FabXol3 (gray) and FabXol2 (pink) structures, Arg81 forms hydrogen bonds with Asn156. In the FabXol3 structure, Arg81 contacts Pro158, while Leu158 is partially disordered in the FabXol2 structure. Arg83 does not form any crystal packing interactions. (*b*) In the FabXol3 structure, CDRH1 and CDRH3 residues (gray) contact the V_L_ domain of a symmetry-related molecule (green). Hydrogen bonds are depicted by black lines. (*c*) Asp55 (CDRH2) forms a salt bridge with Lys211 from the Cκ domain of a symmetry-related molecule (blue).

The packing interactions that involve V_L_ domain residues 81 and 83 in the FabXol and FabXol1 structures are substantially different in the FabXol2 and FabXol3 structures. In contrast to Ser81, which contacts Ser31 (CDRH1) and Tyr54 (CDRH2), Arg81 instead forms hydrogen bonds with Asn156 (Cκ, symmetry-related molecule) (Fig. 4 ▸ *a*). In FabXol3, Arg81 contacts Pro158 (Cκ), while Leu158 is partially disordered in FabXol2. Furthermore, and in contrast to Gln83, which contacts Tyr33 (CDRH1), Tyr54 (CDRH2) and His101 (CDRH3) in the FabXol and FabXol1 structures, Arg83 does not participate in any crystal packing interactions in the FabXol2 and FabXol3 structures (Fig. 4 ▸ *a*). As the overall structures of FabXol2 and FabXol3 are similar, further discussion will be limited to the FabXol3 structure, which was solved at higher resolution (1.45 Å for FabXol3 compared with 2.05 Å for FabXol2).

In the FabXol3 structure, CDRH1 and CDRH3 residues contact the V_L_ domain of one symmetry-related molecule at an interface that includes hydrogen bonds between Ser31 (CDRH1) and Ser69, between Tyr27 (CDRH1) and Tyr57, between Tyr27 and Asp34, between Ser100 (CDRH3) and Asp30, between Phe103 (CDRH3, main chain) and Thr73, and between Gly104 (CDRH3, main chain) and Asp74 (Fig. 4 ▸ *b*). On the other hand, Asp55 (CDRH2) forms a salt bridge with Lys211 from the Cκ domain of a different symmetry-related Fab, and together with Gly56 (CDRH2) packs against Pro117 and Ser118 (Fig. 4 ▸ *c*).

The FabXol3 CDRH1 and CDRH3 conformations are markedly different to those in the FabXol, scFvXol and FabXol1 structures; the nature and implications of these conformational differences are discussed later.

### 3.7. Conformational diversity in the CDRs: comparison of unbound and bound Fab structures   

In the FabXol, scFvXol and FabXol1^1^ structures, and in the molecules FabXol1^2*A*^ and FabXol1^2*B*^, the heavy-chain CDRs adopt similar conformations (Figs. 1 ▸ *b*, 1 ▸ *d*, 2 ▸ *d* and 3 ▸ *b*). However, substantial conformational diversity is observed for CDRH1 and CDRH3 in molecules FabXol1^2*C*^ and FabXol1^2*D*^, and in FabXol3.

**Figure 5:**
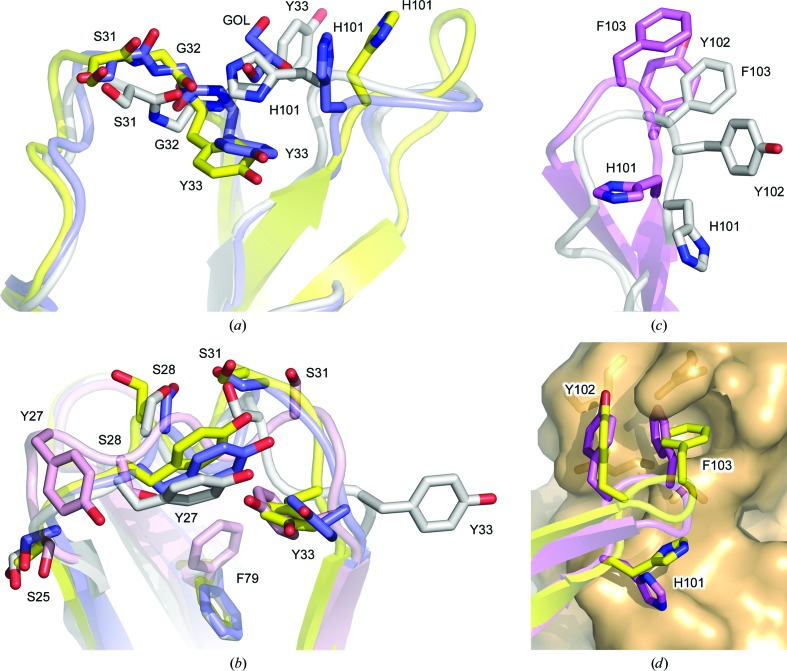
Conformational diversity in the omalizumab CDRs. (*a*) In molecule FabXol1^2*C*^ (blue), the binding of a glycerol molecule (GOL) alters the position of Tyr33 (CDRH1), which adopts a similar position to that in the IgE-Fc-bound Fab (yellow; Davies *et al.*, 2017 ▸). The FabXol^1^ structure (gray) is shown for comparison. (*b*) Compared with the FabXol1 structure (gray), molecule FabXol1^2*C*^ from the FabXol1^2^ structure (blue) and FabXol3 from the complex with IgE-Fc (yellow), CDRH1 adopts a conformation in the unbound FabXol3 structure (pink) that alters the positions of Tyr27 and Ile29 (CDRH1). Phe79 also adopts a different position. By contrast, Tyr33 (CDRH1) adopts a similar position in FabXol1^2*C*^ (blue), unbound FabXol3 (pink) and FabXol3 bound to IgE-Fc (yellow). Tyr33 adopts a substantially different position in FabXol^1^ (gray). (*c*) In the FabXol3 structure (pink), CDRH3 adopts a different conformation compared with that in the FabXol^1^ structure (gray). (*d*) The conformation adopted by CDRH3 in the unbound FabXol3 structure (pink) is similar to that in the FabXol3–IgE-Fc complex (yellow). The surface of the C∊3 domain from the complex is colored orange (Davies *et al.*, 2017 ▸).

In molecules FabXol1^2*C*^ and FabXol1^2*D*^, a glycerol molecule occupies a structurally equivalent position to Ser378 and Gly379 from the C∊3 domain in the complex between the omalizumab-derived Fab and IgE-Fc (Davies *et al.*, 2017 ▸), altering the position of Tyr33 (CDRH1), which adopts a similar position to that in the IgE-Fc-bound Fab (Fig. 5 ▸ *a*). The conformations of Ser31 (CDRH1) and Gly32 (CDRH1) are also similar to those in the complex, presumably due to the conformational change involving Tyr33. In the complex with IgE-Fc, Gly32 and Tyr33 from CDRH1 contribute to the interface with the C∊3 domain, packing against Ala377 and Ser378. The glycerol molecule, close to Tyr33, also causes the His101 (CDRH3) side chain to adopt a different position (Fig. 5 ▸ *a*); however, the overall conformation of CDRH3 is otherwise similar to that in the unbound FabXol, scFvXol and FabXol1^1^ structures and in the molecules FabXol1^2*A*^ and FabXol1^2*B*^.

In FabXol3, residues Ser25–Gly32 (CDRH1) adopt a markedly different conformation compared with the other unbound and bound Fab structures, which alters the positions of Tyr27 and Ile29; the Phe79 side chain, adjacent to CDRH1, also adopts a different position (Fig. 5 ▸ *b*). On the other hand, Tyr33 adopts a similar position to that in the FabXol1^2*C*^ and FabXol1^2*D*^ molecules and the bound Fab structures. Comparison of the FabXol3 structure with the structure of the complex with IgE-Fc (Davies *et al.*, 2017 ▸) reveals that the positions adopted by Ser25–Ser31, and Tyr33 in FabXol3 would not preclude an interaction with the C∊3 domain; however, Gly32 would clash with Ser378. This particular CDRH1 conformation thus appears to be incompatible with IgE binding. By contrast, in FabXol3, CDRH3 adopts a strikingly different conformation compared with the other Fab structures reported here (Fig. 5 ▸ *c*). In these Fab structures, the CDRH3 conformation is incompatible with IgE binding due to steric clashes with the C∊3 domain. However, the CDRH3 conformation in the unbound FabXol3 structure is similar to the conformation adopted by CDRH3 in the FabXol3/IgE-Fc complex (Davies *et al.*, 2017 ▸; Fig. 5 ▸ *d*); a conformational change in the CDRH3 main chain causes a dramatic rearrangement in the positions of side-chain residues, particularly His101, Tyr102 and Phe103, which contact the C∊3 domain in the complex.

In contrast to the structural diversity displayed by CDRH1 and CDRH3, the conformation of CDRH2 is conserved in the unbound Fab and scFv structures, and in the complexes of the omalizumab Fab with the constrained Fc∊3-4 molecule (Pennington *et al.*, 2016 ▸) and of FabXol3 with IgE-Fc (Davies *et al.*, 2017 ▸). Like CDRH2, the light-chain CDR conformations are also conserved; similar conformations are adopted in the 12 independent views reported here and in other unbound Fab structures (Jensen *et al.*, 2015 ▸; Wright *et al.*, 2015 ▸), which are similar to those in the complexes between the omalizumab Fab and the constrained Fc∊3-4 molecule (Pennington *et al.*, 2016 ▸) and between FabXol3 and IgE-Fc (Davies *et al.*, 2017 ▸). Nevertheless, the FabXol2 and FabXol3 crystal structures show substantial conformational diversity in the heavy-chain CDRs, and together with the FabXol1^2^ structure reveal how conformations compatible with IgE binding are adopted in the unbound Fab.

### 3.8. Interaction of the omalizumab-derived Fabs and scFv with IgE-Fc in solution   

The aim of our mutagenesis strategy was to disrupt the crystal packing interactions observed in the wild-type omalizumab (FabXol) crystal structures, without mutating the CDR residues responsible for IgE-Fc binding and significantly affecting the affinity for IgE-Fc. We have previously demonstrated that the kinetics of the interaction between omalizumab and IgE-Fc are biphasic, with one high-affinity (∼1 n*M*) and one lower-affinity (∼30 n*M*) interaction (Davies *et al.*, 2017 ▸), and that FabXol3 has a slightly higher affinity for IgE-Fc than FabXol (wild-type omalizumab Fab) and intact omalizumab (Davies *et al.*, 2017 ▸).

We used surface plasmon resonance analysis to characterize further the interaction between IgE-Fc and the omalizumab-derived Fab and scFv constructs. As we have shown previously, at the highest concentration tested (100 n*M* IgE-Fc), the omalizumab-derived Fabs and scFv all display the same mode of interaction with IgE-Fc, *i.e.* a biphasic model with one higher-affinity and one lower-affinity binding interaction (Davies *et al.*, 2017 ▸). When these data were normalized to have the same maximum binding values, it was found that the association rates were similar to those for intact omalizumab (Davies *et al.*, 2017 ▸; Table 5 ▸). However, a statistically significant trend of increasingly slower dissociation rates was observed: the dissociation rate for the omalizumab-derived Fab (FabXol) is slower than that for intact omalizumab, FabXol2 has a slower dissociation rate than FabXol and FabXol3 is even slower, while the scFvXol dissociation rate is the slowest of all (Table 5 ▸ and Supplementary Fig. S1).

## 4. Discussion   

After unsuccessful attempts to crystallize the complex between the Fab fragment of the therapeutic anti-IgE omalizumab and IgE-Fc, and the Fc∊3-4 region, we designed a mutagenesis strategy to disrupt the substantial, and recurring, crystal packing interactions observed in different omalizumab Fab structures. We targeted crystal packing interactions at two different interfaces. The first interface comprised hydrogen bonds between an edge β-strand from the Cγ1 domain (β-strand G; Lys214–Lys218) and a short segment of β-strand structure in the Cκ domain CD loop (Leu158–Ser160). The second interface involved the omalizumab heavy-chain CDRs and V_L_ domain AB, C′′D and EF loops and Cκ domain DE loop. Our mutations were designed to disrupt these packing interactions without significantly affecting the affinity of omalizumab for IgE, and as such were distal to the antigen-binding CDRs.

Packing interactions similar to that between the Cγ1 domain edge β-strand (strand G) and the Cκ domain CD loop are found in a number of other crystal structures containing Fab fragments (see, for example, Hall *et al.*, 2016 ▸; Lee *et al.*, 2017 ▸; Li *et al.*, 2009 ▸; Sickmier *et al.*, 2016 ▸). Indeed, a variety of packing interactions involving hydrogen-bond networks between β-strands have been detected in crystal structures of intact antibodies and their fragments (Edmundson *et al.*, 1999 ▸; Wingren *et al.*, 2003 ▸), including antiparallel arrangements between edge strands in Cλ and Cγ1 domains (see, for example, Faber *et al.*, 1998 ▸), V_H_ domains (see, for example, Harris *et al.*, 1998 ▸) and V_L_ domains (see, for example, Bourne *et al.*, 2002 ▸).

We mutated Leu158 from the omalizumab Cκ domain CD loop to proline (omalizumab-derived mutant FabXol1) to disrupt the interface with strand G from the Cγ1 domain, and although this was achieved, the FabXol1 molecule still crystallized preferentially, in different packing arrangements stabilized in part by the presence of Pro158.

We next targeted the crystal packing interactions between the omalizumab CDRs and V_L_ and Cκ domain framework residues (V_L_ domain AB, C′′D and EF loops and Cκ domain DE loop) from symmetry-related molecules. We mutated Ser81 and Gln83 from the omalizumab V_L_ domain EF loop to arginine and created two omalizumab-derived mutants: FabXol2 contained the Ser81Arg and Gln83Arg mutations, while FabXol3 additionally contained the Leu158Pro mutation. The IgE-Fc protein was successfully crystallized in complex with both FabXol2 and FabXol3, and the 3.7 Å resolution crystal structure of the FabXol3/IgE-Fc complex was recently reported (Davies *et al.*, 2017 ▸). Engineering the Ser81Arg and Gln83Arg mutations in the V_L_ domain of the omalizumab Fab clearly disrupted the interactions seen in the FabXol structure, but these residues also formed new packing interactions in the FabXol2 and FabXol3 structures that were seen when these molecules were crystallized alone. Presumably, however, these packing contacts were collectively weaker than those in either FabXol or FabXol1, since they were unable to compete with the pre-formed Fab/IgE-Fc complexes and their crystallization.

Unbound IgE-Fc adopts an acutely bent conformation, in which the C∊2 domains fold back against the Fc∊3-4 region (Doré *et al.*, 2017 ▸; Holdom *et al.*, 2011 ▸; Wan *et al.*, 2002 ▸). IgE-Fc is more acutely bent in the crystal structure of the sFc∊RIα/IgE-Fc complex (Holdom *et al.*, 2011 ▸), less acutely bent when in complex with sCD23 (Dhaliwal *et al.*, 2017 ▸), partially bent when in complex with FabXol3 (Davies *et al.*, 2017 ▸) and fully extended in the complexes with the anti-IgE Fabs a∊Fab and 8D6 (Chen *et al.*, 2018 ▸; Drinkwater *et al.*, 2014 ▸); these structures demonstrate that IgE-Fc is conformationally dynamic. However, despite this flexibility, IgE adopts a predominantly bent conformation in solution (Beavil *et al.*, 1995 ▸; Davis *et al.*, 1990 ▸; Holowka & Baird, 1983 ▸; Holowka *et al.*, 1985 ▸; Hunt *et al.*, 2012 ▸; Zheng *et al.*, 1991 ▸, 1992 ▸). The propensity for IgE-Fc to adopt such a bent conformation might account for the selective crystallization of the omalizumab Fab and the omalizumab-derived mutant FabXol1. Bending of IgE-Fc, from the partially bent conformation observed in the FabXol3/IgE-Fc complex to the acutely bent structure, would disrupt one of the omalizumab binding sites on the C∊3 domain. In the FabXol3/IgE-Fc complex, Arg81 and Arg83 from one FabXol3 molecule contact one of the C∊2 domains, in addition to the omalizumab binding site on the C∊3 domain. This additional interaction might stabilize the partially bent conformation in the complex.

In IgE-Fc and Fc∊3-4, the C∊3 domains adopt a range of conformations relative to one another, from closed to open (Chen *et al.*, 2018 ▸; Cohen *et al.*, 2014 ▸; Davies *et al.*, 2017 ▸; Dhaliwal *et al.*, 2012 ▸, 2014 ▸, 2017 ▸; Doré *et al.*, 2017 ▸; Drinkwater *et al.*, 2014 ▸; Garman *et al.*, 2000 ▸; Holdom *et al.*, 2011 ▸; Jabs *et al.*, 2018 ▸; Wan *et al.*, 2002 ▸; Wurzburg & Jardetzky, 2009 ▸; Wurzburg *et al.*, 2000 ▸; Yuan *et al.*, 2013 ▸); this conformational diversity is crucial for the allosteric regulation of IgE binding to its receptors, Fc∊RI and CD23 (Borthakur *et al.*, 2012 ▸; Dhaliwal *et al.*, 2012 ▸). The flexibility of the C∊3 domains could account for our failure to crystallize the complex between the omalizumab Fab and the unconstrained Fc∊3-4 molecule, which lacks the C∊2 domains. Notably, the reported omalizumab Fab complex (Pennington *et al.*, 2016 ▸) is with an Fc∊3-4 molecule that contains an engineered disulfide bond, which locks the C∊3 domains into a closed conformation, thus reducing the overall flexibility of the complex.

Fab fragments are invaluable tools as chaperone proteins for crystallization, and are used for their ability to trap different conformations or reduce flexibility in the target protein (Bukowska & Grütter, 2013 ▸; Griffin & Lawson, 2011 ▸; Rasmussen *et al.*, 2007 ▸; Sun *et al.*, 2018 ▸; Tamura *et al.*, 2019 ▸; Uysal *et al.*, 2009 ▸). However, in our case, crystallization trials of our conformationally flexible target protein, IgE-Fc, in complex with the Fab fragment of the therapeutic anti-IgE antibody omalizumab resulted in the disruption of pre-formed complexes and selective crystallization of the Fab alone.

Here, we have described a successful mutagenesis strategy in which framework regions of the omalizumab Fab were engineered to disrupt recurring crystal packing interactions in the Fab crystal structures, without significantly altering the stability of the Fab, nor its affinity for IgE-Fc. Although disrupting the hydrogen-bond-mediated interactions between β-strands did not prevent selective crystallization of the Fab, the recurring interface between the light chain and CDRs was disrupted by introducing bulkier residues through point mutations in the light-chain framework regions.

This approach, of introducing point mutations distal to the antigen-binding CDRs to disrupt undesired crystal packing interactions, could assist in the structure determination of Fabs in complex either with similarly conformationally flexible, or indeed inflexible, target proteins.

## Supplementary Material

PDB reference: omalizumab Fab, crystal form I, 6tcm

PDB reference: crystal form II, 6tcn

PDB reference: Leu158Pro light-chain mutant, crystal form I, 6tco

PDB reference: crystal form II, 6tcp

PDB reference: Ser81Arg and Gln83Arg light-chain mutant, 6tcq

PDB reference: Ser81Arg, Gln83Arg and Leu158Pro light-chain mutant, 6tcr

PDB reference: omalizumab scFv, 6tcs

Supplementary Figure S1. DOI: 10.1107/S2053230X20001466/ow5019sup1.pdf

## Figures and Tables

**Table 1:** Data-processing and refinement statistics for FabXol^1^, FabXol^2^ and scFvXol Values in parentheses are for the outer shell.

	FabXol^1^	FabXol^2^	scFvXol
Data processing			
Space group	*P*2_1_2_1_2_1_	*P*12_1_1	*P*3_1_21
*a*, *b*, *c* (Å)	65.38, 73.56, 141.10	85.29, 73.57, 87.10	73.91, 73.91, 117.80
β (°)		116.58	
Resolution (Å)	65.38–1.85 (1.89–1.85)	77.89–2.30 (2.42–2.30)	64.01–2.30 (2.38–2.30)
Completeness (%)	99.9 (99.9)	99.5 (96.9)	99.8 (99.9)
Multiplicity	7.2 (6.9)	3.7 (3.2)	5.5 (5.2)
Mean *I*/σ(*I*)	4.3 (1.7)	9.0 (1.8)	6.2 (2.5)
CC_1/2_	0.99 (0.413)	0.993 (0.631)	0.974 (0.597)
*R*_p.i.m._	0.078 (1.101)	0.060 (0.454)	0.121 (1.064)
*R*_merge_	0.192 (2.641)	0.099 (0.674)	0.259 (2.248)
Wilson *B* factor (Å^2^)	22.8	44.0	24.6
Refinement			
*R*_work_/*R*_free_ † (%)	16.77/19.03	18.78/22.58	17.80/20.62
No. of reflections	57943	42804	16902
R.m.s. deviations			
Bond lengths (Å)	0.014	0.002	0.004
Bond angles (°)	1.334	0.535	0.683
Coordinate error (Å)	0.18	0.30	0.20
No. of atoms			
Protein	3357	6468	1751
Solvent	314	246	78
Other	70‡	62§	14¶
Average *B* factor (Å^2^)			
Protein	27.81	47.55	33.68
Solvent	40.07	41.26	38.72
Other	51.82‡	62.02§	53.20¶
Ramachandran plot			
Favored (%)	97.79	97.07	96.98
Allowed (%)	2.21	2.82	3.02

† The *R*_free_ set comprises 5% of the reflections.

‡ 2-Methyl-2,4-pentanediol, glycerol and phosphate.

§ Ethylene glycol, polyethylene glycol, Tris and sulfate.

¶ Polyethylene glycol.

**Table 2:** Data-processing and refinement statistics for FabXol1^1^, FabXol1^2^, FabXol2 and FabXol3 Values in parentheses are for the outer shell.

	FabXol1^1^	FabXol1^2^	FabXol2	FabXol3
Data processing				
Space group	*C*222_1_	*P*2_1_2_1_2_1_	*P*2_1_2_1_2_1_	*P*2_1_2_1_2_1_
*a*, *b*, *c* (Å)	94.46, 116.84, 181.16	80.11, 162.04, 164.43	44.03, 96.61, 103.51	43.72, 96.25, 103.30
Resolution (Å)	47.23–1.80 (1.83–1.80)	82.21–2.50 (2.55–2.50)	28.08–2.05 (2.11–2.05)	33.37–1.45 (1.53–1.45)
Completeness (%)	99.9 (99.9)	99.9 (99.8)	99.7 (96.3)	99.6 (97.6)
Multiplicity	10.0 (10.3)	6.7 (6.7)	7.5 (4.1)	6.8 (4.4)
Mean *I*/σ(*I*)	15.9 (1.7)	8.9 (1.9)	7.5 (1.7)	14.8 (2.7)
CC_1/2_	0.999 (0.596)	0.984 (0.562)	0.990 (0.608)	0.997 (0.832)
*R*_p.i.m._	0.031 (0.513)	0.105 (0.602)	0.075 (0.466)	0.031 (0.250)
*R*_merge_	0.093 (1.572)	0.254 (1.464)	0.197 (0.889)	0.079 (0.475)
Wilson *B* factor (Å^2^)	26.8	13.2	17.4	11.2
Refinement				
*R*_work_/*R*_free_ † (%)	16.76/19.22	21.23/23.88	17.35/22.14	16.60/18.36
No. of reflections	92661	74660	28385	77571
R.m.s. deviations				
Bond lengths (Å)	0.011	0.002	0.008	0.016
Bond angles (°)	1.148	0.529	0.954	1.482
Coordinate error (Å)	0.21	0.34	0.23	0.14
No. of atoms				
Protein	6568	12844	3273	3319
Solvent	498	299	304	341
Other	102‡	128§	6¶	51††
Average *B* factor (Å^2^)				
Protein	32.47	36.23	23.11	19.36
Solvent	38.84	30.32	30.69	31.24
Other	56.02‡	61.11§	45.81¶	34.92††
Ramachandran plot				
Favored (%)	97.62	97.12	97.70	98.22
Allowed (%)	2.38	2.88	2.30	1.78

† The *R*_free_ set comprises 5% of the reflections.

‡ Ethylene glycol and sulfate.

§ Glycerol, polyethylene glycol and sulfate.

¶ Glycerol.

†† Ethylene glycol and polyethylene glycol.

**Table 3:** Nomenclature for the omalizumab-derived Fabs and scFv

Construct	Sequence	Structure	No. of molecules in the asymmetric unit
FabXol	Wild type	FabXol^1^	1: FabXol^1^
		FabXol^2^	2: FabXol^2*A*^, FabXol^2*B*^
FabXol1	Leu158Pro†	FabXol1^1^	2: FabXol1^1*A*^, FabXol1^1*B*^
		FabXol1^2^	4: FabXol1^2*A*^, FabXol1^2*B*^, FabXol1^2*C*^, FabXol1^2*D*^
FabXol2	Ser81Arg†, Gln83Arg†	FabXol2	1: FabXol2
FabXol3	Ser81Arg†, Gln83Arg†, Leu158Pro†	FabXol3	1: FabXol3
scFvXol	Wild type†	scFvXol	1: scFvXol

† Mutation in the Fab light chain.

‡ The V_L_ and V_H_ domains are linked by a (Gly_4_Ser)_4_ linker.

**Table 4:** Thermal stabilities of the omalizumab-derived Fabs

	*T*_m_ (°C)
FabXol	79.9 ± 0.5
FabXol1	79.0 ± 0.7
FabXol2	77.1 ± 0.5
FabXol3	78.8 ± 0.4

**Table 5:** Kinetics of omalizumab, the omalizumab-derived Fabs and scFv binding to IgE-Fc

Molecule immobilized	*k*_on1_ (*M* ^−1^ s^−1^)	*k*_on2_ (*M* ^−1^ s^−1^)	*k*_off1_ (s^−1^)	*k*_off2_ (s^−1^)
Omalizumab	3.3 × 10^5^	2.9 × 10^5^	7.0 × 10^−4^	1.2 × 10^−2^
FabXol	5.7 × 10^5^	4.4 × 10^5^	5.6 × 10^−4^	1.2 × 10^−2^
FabXol2	5.1 × 10^5^	3.3 × 10^5^	4.5 × 10^−4^	1.1 × 10^−2^
FabXol3	9.7 × 10^5^	2.7 × 10^5^	3.3 × 10^−4^	9.0 × 10^−3^
scFvXol	6.9 × 10^5^	3.1 × 10^5^	2.9 × 10^−4^	8.7 × 10^−3^
