# Supplementary material for: Engineering the Fab fragment of the anti-IgE omalizumab to prevent Fab crystallization and permit IgE-Fc complex crystallization
Source: Acta Crystallogr F Struct Biol Commun. 2020 Mar 2;76(Pt 3):116–29. doi: 10.1107/S2053230X20001466 (PMC7057348; doi:10.1107/S2053230X20001466)
Supplement: Supplementary file 1 [file f-76-00116-sup1.pdf]

STRUCTURAL BIOLOGY COMMUNICATIONS

Volume 76 (2020)

Supporting information for article:

Engineering the Fab fragment of the anti-IgE omalizumab to prevent Fab crystallization and permit IgE-Fc complex crystallization

Alkistis N. Mitropoulou, Tom Ceska, James T. Heads, Andrew J. Beavil, Alistair J. Henry, James M. McDonnell, Brian J. Sutton and Anna M. Davies

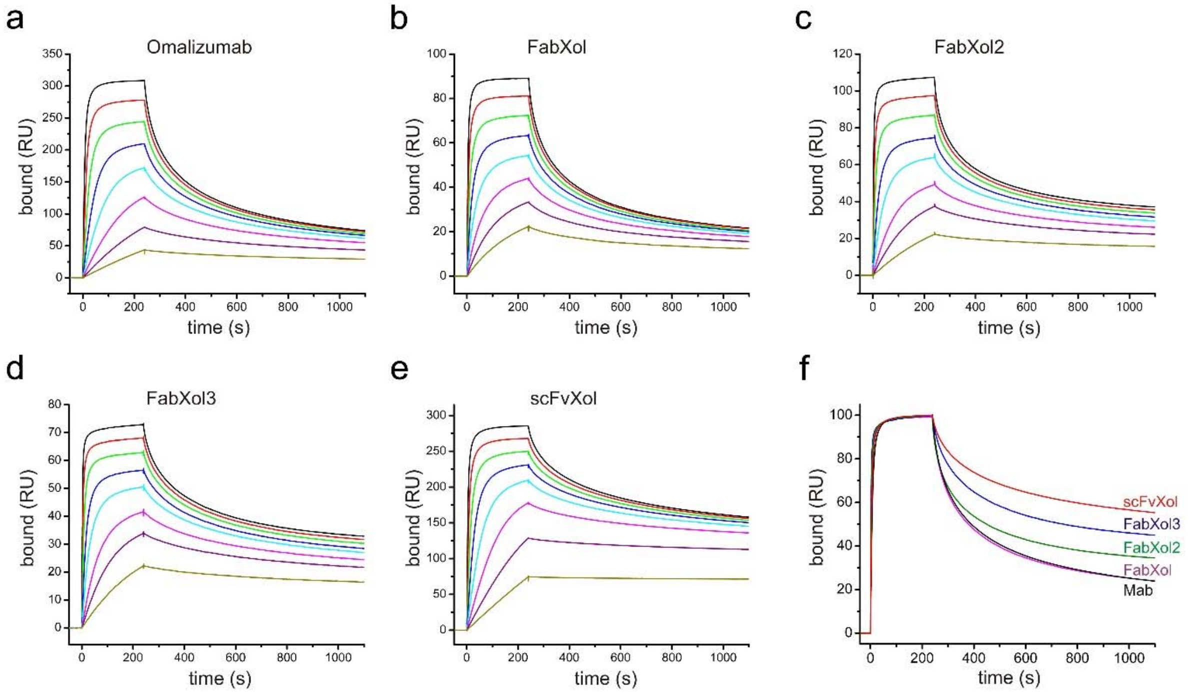

Figure S1 SPR sensorgrams of IgE-Fc binding to the following immobilized antibody constructs: (*a*) omalizumab, (*b*) FabXol (wild-type omalizumab Fab), (*c*) FabXol2, (*d*) FabXol3, and (*e*) scFvXol. IgE-Fc was injected over the surface at concentrations of 0.4nM (dark blue), 0.8nM (brown), 1.6nM (purple), 3.2nM (magenta), 6.4nM (cyan), 13nM (blue), 25nM (green), 50nM (red), and 100nM (black). (*f*) A comparison of normalized SPR measurements to assess the binding of 100nM IgE-Fc to omalizumab (Mab, black), FabXol (purple), FabXol2 (green), FabXol3 (blue), and scFvXol (red). The association rates of IgE-Fc to these different constructs are similar, but clear differences in off-rates can be observed.
